# Combined effects of elevated epilimnetic temperature and metalimnetic hypoxia on the predation rate of planktivorous fish

**DOI:** 10.1093/plankt/fbz048

**Published:** 2019-10-09

**Authors:** Piotr Maszczyk, Ewa Babkiewicz, Krzysztof Ciszewski, Kamil Dabrowski, Przemysław Dynak, Karol Krajewski, Paulina Urban, Marcin Żebrowski, Wojciech Wilczynski

**Affiliations:** 1 DEPARTMENT OF HYDROBIOLOGY, FACULTY OF BIOLOGY, BIOLOGICAL AND CHEMICAL RESEARCH CENTRE AT UNIVERSITY OF WARSAW, ŻWIRKI I WIGURY 101, 02-089 WARSAW, POLAND; 2 LABORATORY OF FUNCTIONAL AND STRUCTURAL GENOMICS, CENTER OF NEW TECHNOLOGIES, UNIVERSITY OF WARSAW, BANACHA 2C, WARSAW 02-097, POLAND; 3 COLLEGE OF INTER-FACULTY INDIVIDUAL STUDIES IN MATHEMATICS AND NATURAL SCIENCES, UNIVERSITY OF WARSAW, BANACHA 2C, 02-097 WARSAW, POLAND

**Keywords:** *Daphnia*, hypoxia, metalimnetic refuge, planktivorous fish, temperature

## Abstract

Increased temperature in the epilimnion and hypoxia in the metalimnion of a lake would result in an increase of positive-size-selective fish predation on zooplankton and in turn in a decrease of mean body size in zooplankton populations and communities. We tested this hypothesis in four types of experiments with juvenile rudd (*Scardinius erythrophthalmus*) foraging on *Daphnia longispina* in an indoor twin column tank system. In each experiment of the first three types, one column contained one of three types of experimental treatments differing from the control treatment (in the other column) by the following: (i) elevated temperature in the epilimnion, (ii) hypoxia in the metalimnion and (iii) simultaneous elevated temperature in the epilimnion and hypoxia in the metalimnion. In the fourth type of experiment, the gradients of temperature and oxygen concentration in both columns were the same, but prior to the experiments, *Daphnia* and fish in the control treatment were acclimated to normoxia and, in the experimental treatment, to hypoxia. The results confirmed our hypothesis, since the predation rate of fish was greater in each of the first three experimental treatments than in the control. We did not detect an effect of the acclimation to hypoxia on the predation rate of the fish.

## INTRODUCTION

Numerous studies have demonstrated that the mean body size in populations and communities across species in a variety of taxonomic groups of aquatic ectotherms, including freshwater zooplankton, is positively correlated with latitude, and therefore, inversely correlated with temperature (Poulin, 1995; Gillooly and Dodson, 2000; Havens *et al*., 2015; see Maszczyk and Brzezinski, 2018 for review). The trend toward a smaller body size at elevated ambient temperatures is also apparent in other geographical clines (i.e. along altitude and depth), either in marine or in freshwater environments (Green, 1995; Mauchline, 1972; Maszczyk and Brzezinski, 2018). Moreover, the negative correlation between temperature and the proportion of large-bodied species has long been recognized in the seasonally variable size structure of the zooplankton community, in which large-bodied species tend to decline during summer (Sommer *et al.* 1986; Dupuis and Hann, 2009). This correlation has also been observed in the long-term changes in the mean body size of zooplankton communities (Adrian and Deneke, 1996; Benndorf *et al.*, 2001; Gauthier *et al*., 2014; Sastri *et al*., 2014), which suggests that it might also be apparent with the increasing mean temperature due to global warming.

One of the possible explanations of the body size distribution pattern in zooplankton populations and communities is that a higher water temperature leads to increased predation by fish, which selectively forage on larger zooplankton. It could be expected that the increased intensity of positive size selective predation on zooplankton might lead to a reduction of their mean body size at population and community levels (Brooks and Dodson 1965; Gliwicz and Wrzosek 2008) due to both increasing mortality (Brooks and Dodson 1965; Gliwicz and Wrzosek 2008) and the forcing of phenotypic changes to the body size of individuals (Macháček, 1991; Maszczyk and Bartosiewicz, 2012). Large-bodied cladoceran order *Daphnia*, a key zooplankton organism in the majority of freshwaters, are particularly the most vulnerable. Although various lines of evidence suggest or even indicate that fish predation is a key factor determining the prevalence of small-bodied zooplankton in zooplankton populations and communities in warm lakes (Bernot *et al*., 2006; Jeppesen *et al*., 2010; Iglesias *et al*., 2011; Vadadi-Fülöp *et al*., 2012), it is not clear which mechanisms are responsible for this pattern (Gillooly and Dodson, 2000; Maszczyk and Brzezinski, 2018). One potential mechanism is based on the observation that elevated temperature modifies the shape of the vertical gradients of physical factors, such as temperature and oxygen concentration (Hu and Tessier, 1995; De Stasio *et al*., 1996), which would result in an increased predation rate of planktivorous fish.

Long-term correlative and simulation studies on the effects of elevated temperature on the biophysical parameters of stratified temperate and subtropical lakes indicated that during warm years, the temperature of the epilimnion increases, but the temperature of the hypolimnion usually remains unchanged or is even colder (Robertson and Ragotzke, 1990; De Stasio *et al*., 1996; Straile *et al*., 2003; Kirillin, 2010; Bartosiewicz et al., 2019). This, in turn, can lead to an increased predation rate in warmer water in the epilimnion (Persson, 1986; Bergman, 1987; Linløkken *et al*., 2010; Gliwicz and Maszczyk, 2016), since the thickness of the epilimnion increases and the thickness of the metalimnion (which serves as a refuge for zooplankton) decreases. These predictions were indirectly confirmed by several field studies describing an experiment using an entire lake, which revealed that deepening the thermocline resulted in an increased overall zooplankton biomass, increased reproduction rates and secondary production, as well as in decreased mean body size of zooplankton communities (Gauthier *et al*., 2014; Sastri *et al*., 2014). Although these effects were partially attributed to an increased predation rate of planktivorous fish, due to the specificity of the field experiments, which are constrained by the inability to examine the effect of a single factor by excluding all other factors, the literature still does not provide any direct evidence that deepening the thermocline results in an increased predation rate of planktivorous fish.

Correlative studies on the seasonal effects of elevated temperature on shallow and deep temperate and subtropical lakes also revealed that the oxygen concentration in the metalimnion (and hypolimnion) usually decreases with warming (Hu and Tessier, 1995; Sell, 1998) and that this deficiency strongly correlates with shifts in the zooplankton community from large- to small-bodied zooplankton, which was attributed to increased predation by fish (e.g. Hu and Tessier, 1995). Experimental evidence is less consistent. While three field studies confirmed that decreased meta- and hypolimnetic oxygen concentrations resulted in a shift from large- to intermediate-bodied *Daphnia* (Wright and Shapiro, 1990; Field and Prepas, 1997; Chang *et al*., 2013), a single indoor study (Larsson and Lampert, 2011) revealed the opposite effect, which was attributed to a decreased mortality risk in the presence of metalimnetic hypoxia, because *Daphnia* is more tolerant of lower oxygen concentrations than most pelagic fish species (Weider and Lampert, 1985; Klumb *et al*., 2004; Stanley and Wilson, 2004; Vanderploeg *et al*., 2009a, 2009b). However, the experimental design of the study conducted by Larsson and Lampert (2011) did not allow verification of the possibility that either the fish’s acclimation to hypoxia or the shrinking thickness of the metalimnion (due to the increased temperature of the epilimnion) would increase the mortality risk caused by planktivorous fish at a low oxygen concentration in the metalimnion. Moreover, the study was performed with a deep chlorophyll maximum, which is not the most typical scenario in lakes, and which, in turn, would unduly improve food conditions in the metalimnion for zooplankton, increasing the mean depth selected and decreasing the mortality risk from fish predation.

Although it has been revealed that several *Daphnia* species could tolerate even very low ambient oxygen concentrations (between 1 and 3 mg L^−1^), which are avoided by most pelagic fish (e.g. Vanderploeg *et al*., 2009a), two arguments may suggest that the mortality risk from fish would be greater at a decreased oxygen concentration in the metalimnion. The first argument is that persisting in an environment with a low oxygen concentration has high physiological and in turn demographic costs for zooplankton (Kobayashi and Hoshi, 1984; Hanazato and Dodson, 1995; Larsson and Lampert, 2011; Wilczyński *et al*., 2019). This is consistent with the field observations that maximum zooplankton density during the day is usually in the top part of the metalimnion with relatively high oxygen concentration, even when the light intensity allows fish to forage in this layer (e.g. Vanderploeg *et al*., 2009a). The second argument is based on the observation that a variety of fish species have evolved various behavioral (e.g. dwelling for short time periods in the oxygen-deficient meta- and hypolimnion and foraging there even in highly hypoxic conditions; Vanderploeg *et al*., 2009a, 2009b), anatomical (expanding the gill surface, e.g. Matey *et al*., 2008) and physiological (regulating the gene expression of glycolytic enzymes and hormones; Van Raaij *et al*., 1996; Gracey *et al*., 2001; synthesis of respiratory proteins, i.e. globins; Roesner *et al*., 2006) adaptations of their phenotype response that enable them to cope with hypoxic conditions (e.g. Nikinmaa and Rees, 2005), which would outweigh the adaptations of *Daphnia* to oxygen deficiencies. For instance, contrary to fish, planktonic animals such as *Daphnia* would not be able to produce sufficient amounts of hemoglobin (the only protein responsible for the transport of oxygen in *Daphnia*; Hebert *et al*., 1999), because an increase of its content in the hemolymph results in a reddish body coloration, making *Daphnia* more conspicuous to fish (Confer *et al*., 1978; Wilczyński et al., 2019). Moreover, long-term (duration of few days) acclimation of fish to hypoxia would increase their hunting abilities in hypoxic conditions and thus increase their pressure on zooplankton.

The aim of this study was to test four specific hypotheses concerning the effect of elevated temperature in the epilimnion and hypoxic conditions in the metalimnion on the predation rate of planktivorous fish on *Daphnia*. First, we hypothesize that the predation rate of planktivorous fish increases in a sharper thermocline (resulting from an elevated temperature in the epilimnion), giving fish easier access to the metalimnion, in which the density of *Daphnia* is the highest. Second, that the predation rate of planktivorous fish also increases due to the decreased oxygen concentration in the meta- and hypolimnion. Third, that the combined effect of elevated epilimnetic temperature and metalimnetic hypoxia decreases the metalimnetic refuge to a greater extent, and in turn increases the predation rate to a greater extent, than each of the two conditions alone. Fourth, that the acclimation of planktivorous fish and *Daphnia* to hypoxia increases the predation rate of planktivorous fish on *Daphnia* in the vertical gradients of light intensity, temperature and oxygen concentration (with hypoxic conditions in the meta- and hypolimnion).

**Fig. 1:**
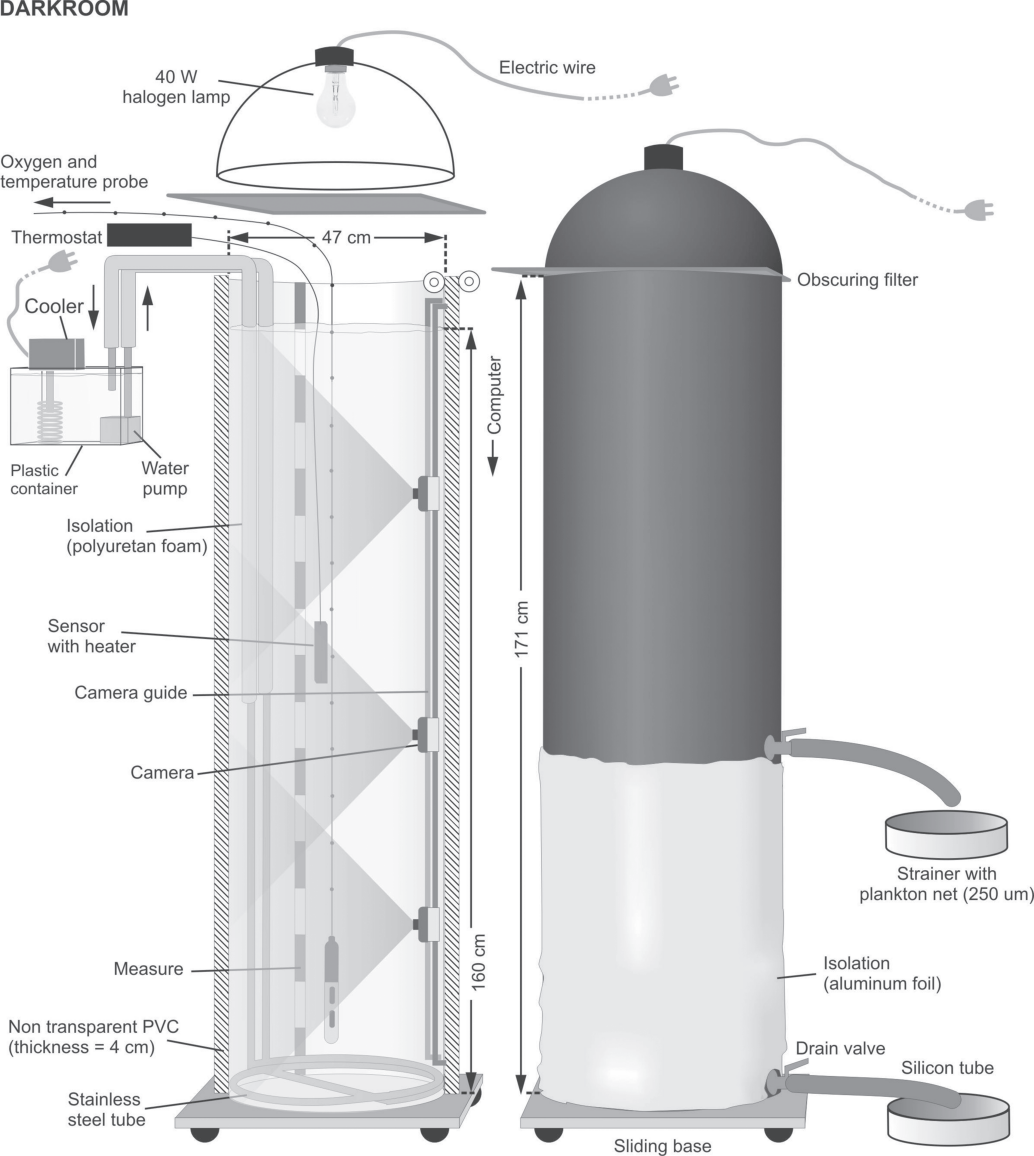
The system comprised twin non-transparent vertical columns used in the experiments.

**Fig. 2:**
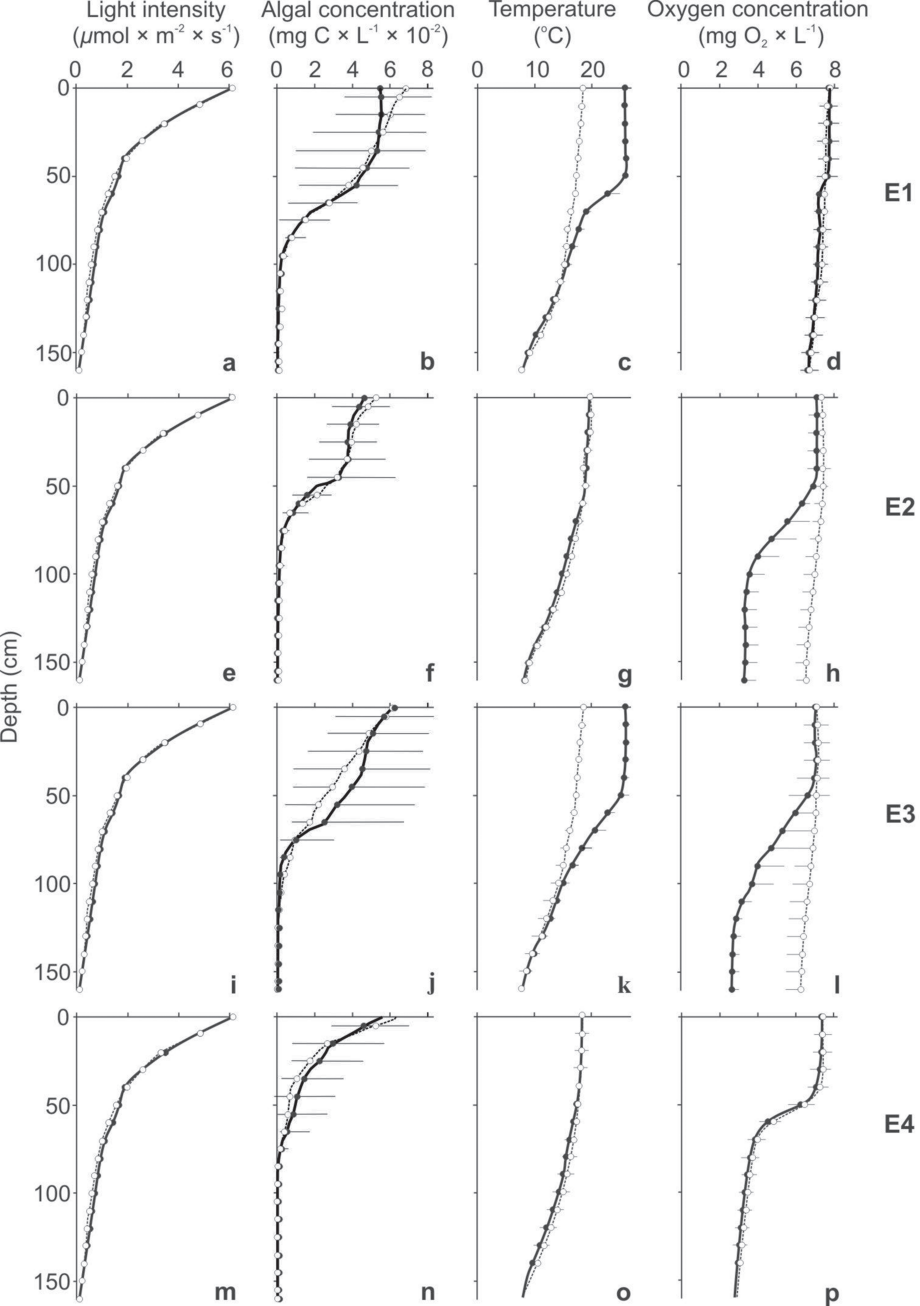
Light intensity (**a**, **e**, **i**, **m**), algal concentration (**b**, **f**, **j**, **n**), temperature (**c**, **g**, **k**, **o**) and oxygen concentration (**d**, **h**, **l**, **p**) gradients in the control (dotted line, mean ± 1 SD) and in one of the four experimental treatments (solid line, mean + 1SD): (1) elevated temperature in the epilimnion (a–d), (2) the presence of hypoxia in the meta- and hypolimnion (e–h), (3) both higher temperature in the epilimnion and hypoxia in the meta- and hypolimnion (i–l) and (4) Daphnia and fish acclimated to hypoxia before the experiments (m–p).

## METHOD

### The approach

To verify the hypotheses, we performed 40 experiments with planktivorous fish (juvenile rudd, *Scardinius erythrophthalmus*). In each of these experiments, a pair of fish was allowed to forage for 10 min on a cohort of 5-day old *Daphnia longispina*. The experimental set-up was composed of twin nontransparent columns (Fig. 1), which allowed us to create the same gradients of light intensity and algal food concentrations in both columns, but different gradients of temperature and oxygen concentrations in each of them (Fig. 2). We performed four types of experiments; each of them was replicated 10 times. In each experiment of the first three types (E1, E2 and E3), the control treatment (high oxygen concentration—normoxia—throughout the water column and temperature gradient from 18°C in the epilimnion to 7°C in the hypolimnion) was in one column and the experimental treatment in the second column, differing from the control by the following: (E1) higher temperature in the epilimnion, (E2) the presence of a low oxygen concentration (hypoxia) in the meta- and hypolimnion and (E3) both higher temperature in the epilimnion and hypoxia in the meta- and hypolimnion. In the fourth type of experiment (E4), the gradients of temperature and oxygen concentration (with meta- and hypolimnetic hypoxia) were the same in both columns, but *Daphnia* and fish were acclimated to normoxia in the control column and to hypoxia in the experimental treatment 2 days before the experiments. Each of the experimental treatments was repeated five times in one column and five times in the second column. Before each of the four types of experiments, *Daphnia* were acclimated for 2 hours to the experimental conditions. At the beginning of the experiments, we monitored the vertical distribution of *Daphnia* and during the experiments, we monitored the vertical distribution of fish using high resolution underwater infra-red cameras (Fig. 1). We counted and measured the remaining *Daphnia* after each experiment to compare the predation rate of the fish in the control and in the experimental treatments.

### Experimental animals

For the experiments, we used juvenile (3.6 ± 0.6 cm) rudd (*S. erythrophthalmus*), a cyprinidae fish from the temperate zone, originating from a hatchery at the Institute of Inland Fisheries (Żabieniec, Poland). Fish in the laboratory culture were fed daily with a small amount of frozen *Chironomidae* larvae and frozen *Daphnia*. Prior to the experiments, they were starved for 24 hours. Two new pairs of naive fish were used in each experiment. Each pair of fish was maintained in a 40 L aquarium 2 days before the experiments, either in hypoxia (in the experimental treatment in E4) or in normoxia (in the remaining cases).

The prey consisted of a single clone (LOR01) of *D. longispina*, originating from the eutrophic Lake Roś (Great Mazurian Lakes, Poland), which is known to be inhabited by various planktivorous fish (Jachner 1991). During our study, *Daphnia* were cultured in 20 glass containers (V = 4 L) in medium with a similar density (30–35 ind L^−1^) and with the same high *Chlamydomonas reinhardtii* food level (1.0 mg C L^−1^) supplied daily. Both fish and *Daphnia* were maintained before the experiments at the same low light intensity (0.2 μmol × m^−2^ s^−1^), photoperiod (16 D:8 N) and temperature (21°C). Both the fish and *Daphnia* were kept in the same laboratory room as the experimental system before the experiments.

### Experimental system

The system consisted of twin identical, vertical, non-transparent plastic (PVC) columns, 0.45 m in diameter and 1.73 m in depth (Fig. 1), similar to those used in the study of Gliwicz *et al*. (2012). The two columns, placed side by side, were illuminated from above with a 20-W lamp placed in nontransparent lampshades isolated from the columns with a semitransparent black filter to produce an illumination gradient with exponentially decreasing intensity from 6.17 ± 0.05 μmol m^−2^ s^−1^ at the surface to 0.00 + 0.00 μmol m^−2^ s^−1^ at the bottom of the water column (Fig. 2). The temperature gradient was created by cooling the bottom water layers and heating the top water layers of each column. The bottom water was cooled by pumping (using a small EHEIM 1000.220 water pump) distilled water from a container with a cooler through insolated (covered with heat-insulating foam at the first 90 cm from the water surface) 1.0 cm diameter stainless steel tubes passing adjacently to the inner wall through the bottom of each column in a closed loop (Fig. 1). The top part of the columns were heated using the ADA-REX ZEFIR water heater with a thermoregulatory capability of 0.1°C submerged at a depth of 80 cm from the water surface of each column either to obtain 26 or 18°C in the epilimnion in the different experimental treatments and in the control (Fig. 2). The temperature was monitored at every 10 cm using a miniaturized waterproof electronic thermometer with a sensor at the end of a cable (Fig. 1). The gradient of oxygen concentration was obtained first by creating hypoxic conditions in the whole water column, then by aerating the top part of the water column. Hypoxic conditions were created (before the temperature gradient was created) by pumping 100% purified nitrogen to the bottom of each of the columns. An air pump (Aqua Lifter AQ-20 Dosing Pump) was used to aerate the top part of the column. The oxygen concentration at every 20 cm of the water columns was monitored with a portable probe (YSI ProODO hand-held dissolved oxygen meter). In the experiments, the algal gradient (from 0.053 ± 0.023 mg C_org._ × L^−1^ at the surface to 0.00 mg C_org._ × L^−1^ at the bottom of the water column; Fig. 2) was created by adding the same amount (1 L at concentration of 1 mg C × L^−1^) of algae under the surface of each of the water columns. The algal gradient was assessed just before the experiments using a portable fluorometer (AquaFluor handheld fluorometer, Turner Designs, USA) with a miniature sensor, which was gently introduced at every 20 cm into each of the columns. Three miniature high resolution underwater cameras (P. P. H. Matar KT-370/540) placed at the depths 35, 85 and 125 cm in each of the columns allowed an accurate assessment to be made of the distribution of *Daphnia* and fish.

### Experimental procedure

Two days before the experiments, both *Daphnia* and fish were transferred to 10 L containers with tap water, which was nitrogenated (for the animals prepared for the experimental treatment in E4) or aerated (for the remaining cases) daily. An hour before the experiments with acclimation to hypoxia, the water in the containers was aerated. In the evening, 12 hours before each experiment, conditioned tap water was pumped to each column (through 0.45 μm pore size filters) from a 500 L barrel with two juvenile rudds. Therefore, the water contained chemical information on predation (fish kairomones). In the experiments with meta- and hypolimnetic hypoxia, hypoxic conditions were created in the bottom part of the column. Then, the bottom water of each treatment was cooled at night. Two hours before the experiments, the top part of each of the water columns was aerated to obtain normoxic conditions and heated to establish the final temperature gradient. Next, the algal gradient was established, the light in each of the columns was switched on and the underwater cameras (connected to a computer) were switched to infrared mode in order to register the depth selection behavior of the *Daphnia* and fish. Then, *Daphnia* (400 individuals, acclimated to normoxia or hypoxia) were gently introduced to the subsurface layer of each of the columns. After 1 hour of *Daphnia* acclimation, just before the experiments, we assessed their distribution by counting individuals twice at each depth (every 20 cm) in each of the columns (dividing the water column into eight sections). Then, the experiment was initiated by introducing two fish (acclimated to hypoxia or normoxia) to each column. The fish were allowed to feed for 10 min. After this time, the light in the columns was switched off, the fish were removed (using a net with a 6 mm mesh, which was later rinsed just in case some *Daphnia* were captured) and the entire volume of 200 L of water from each column was sieved through a 250 μm mesh plankton net. The depth selection behavior of the fish was assessed on the films saved on a computer disk. The distribution of the fish in each experiment was determined by assessing the mean residence time of the fish at three depth layers (0–50, 50–110 and 110–160 cm). Samples of *Daphnia* were preserved in plastic containers with 2% formalin until further analysis.

**Table I:** *Results of the Student’s* t-*test for independent samples comparing* Daphnia *density in each of the sectors (at different depth ranges) between the experimental treatment and control in each of the four types of experiments: (E1) control and treatment with higher temperature in the epilimnion, (E2) control and treatment with a low oxygen concentration (hypoxia) in the meta- and hypolimnion, (E3) control and treatment with both higher temperature in the epilimnion and hypoxia and (E4) control with* Daphnia *and fish acclimated to normoxia and treatment with* Daphnia *and fish acclimated to hypoxia*

Exp. type	Sector # (depth range in centimeters)	Exp. treatment (*n* = 10)		Control (*n* = 10)				95% CI		
		M	SD	M	SD	*t*	*P*	LL	UL	Cohen’s *d*
E1	1 (0–20)	11.79	7.70	12.76	10.61	−0.23	0.818	−9.68	7.74	0.10
	2 (20–40)	12.45	7.10	10.23	6.75	0.72	0.482	−4.28	8.74	0.32
	3 (40–60)	18.24	8.05	15.04	10.96	0.74	0.467	−5.84	12.23	0.33
	4 (60–80)	24.76	10.77	25.61	13.73	−0.15	0.880	−12.44	10.75	0.07
	5 (80–100)	23.07	10.45	26.07	13.19	−0.56	0.580	−14.18	8.18	0.25
	6 (100–120)	5.69	3.41	6.37	5.43	−0.34	0.740	−4.94	3.58	0.15
	7 (120–140)	2.39	2.13	1.60	1.07	1.06	0.304	−0.79	2.38	0.47
	8 (140–160)	0	0	0	0	-	-	-	-	-
E2	1 (0–20)	53.03	20.84	33.32	10.98	2.65	**0.020**	3.70	35.73	1.18
	2 (20–40)	23.10	8.60	23.67	10.83	−0.13	0.898	−9.76	8.62	0.06
	3 (40–60)	10.88	6.66	13.92	1.83	−1.39	0.193	−7.88	1.80	0.62
	4 (60–80)	7.71	6.07	10.00	6.89	−0.79	0.441	−8.39	3.81	0.35
	5 (80–100)	2.79	4.21	9.93	8.69	−2.34	**0.031**	−13.55	−0.72	1.05
	6 (100–120)	2.02	3.02	6.06	5.38	−2.07	0.053	−8.14	0.05	0.93
	7 (120–140)	1.64	2.74	2.58	2.58	−0.80	0.436	−3.45	1.55	0.36
	8 (140–160)	0.29	0.41	0.60	0.61	−1.36	0.192	−0.81	0.17	0.61
E3	1 (0–20)	16.91	8.50	16.46	6.88	0.13	0.898	−6.81	7.72	0.06
	2 (20–40)	27.00	8.75	16.35	8.05	2.83	**0.011**	2.76	18.56	1.27
	3 (40–60)	17.39	5.59	14.61	7.96	0.90	0.378	−3.68	9.24	0.40
	4 (60–80)	15.81	6.66	14.68	6.90	0.38	0.712	−5.23	7.51	0.17
	5 (80–100)	12.37	4.55	16.04	7.48	−1.32	0.202	−9.48	2.15	0.59
	6 (100–120)	5.46	3.71	11.02	6.65	−2.31	**0.033**	−10.62	−0.50	1.03
	7 (120–140)	4.70	4.31	7.18	4.11	−1.32	0.204	−6.44	1.47	0.59
	8 (140–160)	2.80	3.29	3.04	2.74	−0.17	0.866	−3.07	2.61	0.08
E4	1 (0–20)	22.18	6.32	24.60	8.86	−0.71	0.490	−9.65	4.80	0.32
	2 (20–40)	16.89	5.44	16.65	4.32	0.11	0.913	−4.37	4.85	0.05
	3 (40–60)*	12.15	5.58	12.04	5.81	0.04	0.968	−5.25	5.46	0.02
	4 (60–80)	13.25	3.29	15.90	7.66	−1.00	0.335	−8.38	3.09	0.45
	5 (80–100)	13.59	3.25	7.81	7.00	2.37	0.034	0.49	11.06	1.06
	6 (100–120)	10.04	3.18	12.19	3.45	−1.45	0.164	−5.27	0.96	0.65
	7 (120–140)	6.95	4.18	7.24	3.66	−0.16	0.872	−3.98	3.41	0.07
	8 (140–160)	4.36	4.87	2.99	2.09	0.81	0.431	−2.28	5.01	0.36

*In one case (for the comparison of the distributions in E4 in sector 3) the results are from the Mann–Whitney *U* test. Significant differences are marked in bold. *M* – mean; SD – standard deviation; *t* – Student’s *t*-test result; *P* – significance; CI *–* confidence interval; *LL* – lower limit; *UL* – upper limit.

### Data analysis

All of the main statistical analyses were preceded by the basic descriptive statistical analyses including the Shapiro–Wilk test. In order to assess the difference in the distribution of *Daphnia* and fish in each of the sectors of the water column between the control and experimental treatments in each of the four types of experiments separately, we performed a parametric test (Student’s *t*-test for independent samples), and—additionally—Mann–Whitney *U* test. Since the fish distributed only in the two upper sectors (0–50 and 50–90 cm), the data of the fish distribution in the bottom sector (90–160 cm) in each of the four types of the experiments were excluded from the statistical analysis.

The predation rate of the fish was calculated from the difference between the number of *Daphnia* at the beginning and at the end of the experiment divided by the duration of the experiments (12 min) and by the number of foraging fish (2 individuals). To assess the difference in the predation rate between the control and experimental treatment in each of the four types of experiments separately, the Student’s *t*-test for independent samples was performed. Additionally, to assess the difference in the predation rate between experimental treatments, one-way analysis of variance (ANOVA) with least significant difference (LSD) *post-hoc* test was performed. The level of significance was set at *α* = 0.05 for all of the statistics. The statistics were calculated with IBM SPSS Statistics 23 software.

## RESULTS

### The basic descriptive statistics of the measured quantitative variables

In the majority of data sets, the Shapiro–Wilk test was not significant, neither for the data of the distribution of *Daphnia* (Table A1–A4 in the Appendix 1) and fish (Table A5 in the Appendix 1), nor for the data of the predation rate of the fish (Table A6 in the Appendix 1) in the control and in the experimental treatments in each of the four types of experiments. In the remaining cases (Table A1–A6 in the Appendix), the level of skewness ranged from −2 to +2; therefore, it could be assumed that the distributions are not greatly asymmetrical in relation to the average (George and Mallery, 2010). Therefore, for all of the aforementioned data, we assessed the difference in the distribution of *Daphnia* and fish, and the predation rate of the fish between the control and experimental treatment in each of the experimental types (E1–E4) using the parametric test (Student’s *t*-test for independent samples). The only exception was the data for the distribution of *Daphnia* in sector 3 (in a range between 40 and 60 cm) in the fourth experimental treatment, for which the level of skewness was out of the range. Therefore, to assess the difference in the distribution of *Daphnia* in this sector between the control and experimental treatment in E4, we used the nonparametric test (Mann–Whitney *U* test).

### Comparison of Daphnia density between each experimental treatment and the control

**Fig. 3:**
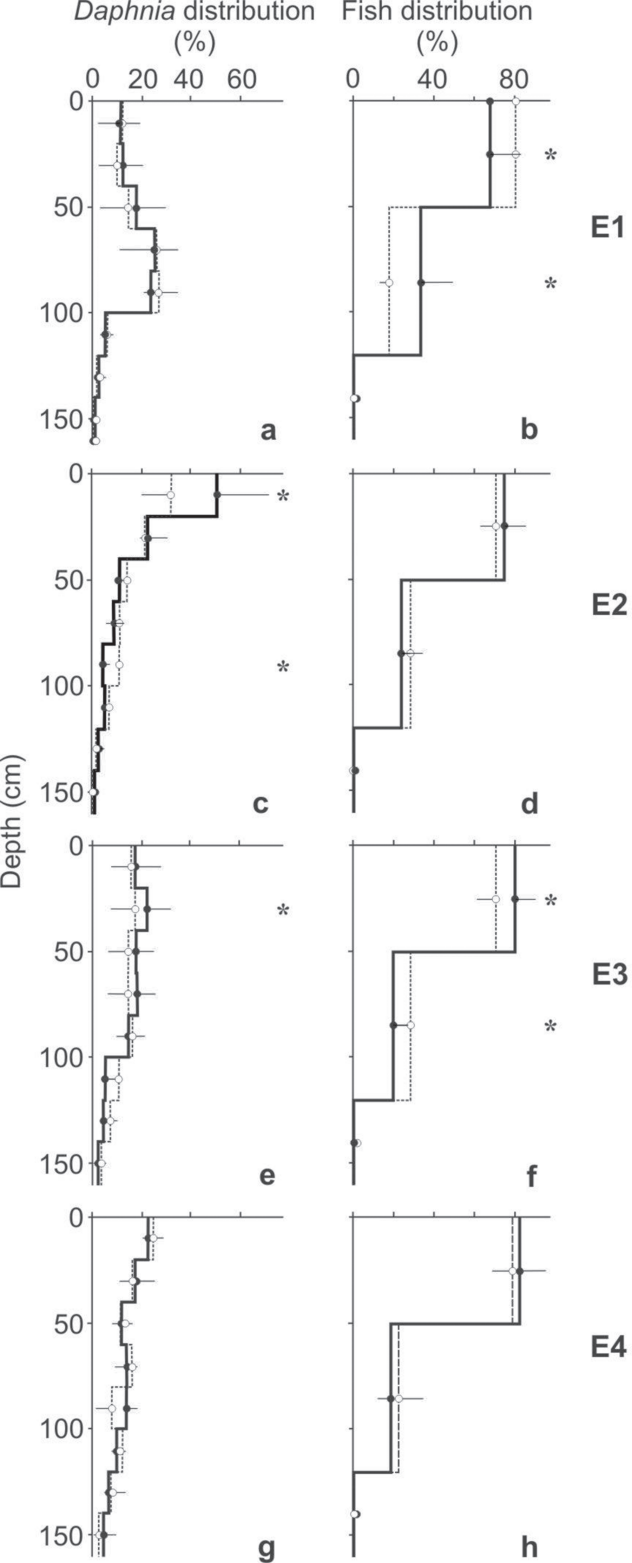
Distribution (mean, %) of Daphnia (**a**, **c**, **e**, **g**) and fish (**b**, **d**, **f**, **h**) in the control (dotted line, mean ± 1 SD) and in one of the four experimental treatments (solid line, mean + 1SD): (1) elevated temperature in the epilimnion (a, b), (2) the presence of hypoxia in the meta- and hypolimnion (c, d), (3) both higher temperature in the epilimnion and hypoxia in the meta- and hypolimnion (e, f) and (4) Daphnia and fish acclimated to hypoxia before the experiments (g, h). Significant differences between the mean depth selected in the control and experimental treatment within each of the four types of experiments for each sector separately are shown as * at *P* < 0.05, all other comparisons within the sectors were not significant (Student’s *t*-test for independent samples or Mann–Whitney *U* test).

Comparing the distribution of *Daphnia* separately for each of the sectors revealed no significant differences between the control and experimental treatment in E1 and E4 (Student’s *t*-test or Mann–Whitney *U* test, Table I; Fig. 3). In E2, differences were found in two sectors. The distribution in sector 1 was greater in the experimental treatment and greater in the control in sector 5 (Student’s *t*-test, Table I; Fig. 3), which suggests that in the presence of meta- and hypolimnetic hypoxia, *Daphnia* selected depths slightly closer to the surface in relation to the control. In E3, differences were also found in two sectors. The distribution in sector 2 was greater in the experimental treatment and greater in the control in sector 6 (Student’s *t*-test, Table I; Fig. 3), which suggest that *Daphnia* selected depths slightly closer to the surface in the presence of both a higher temperature in the epilimnion and meta- and hypolimnetic hypoxia in relation to the control. In each of the four comparisons where significant differences were found (two in E2 and two in E3), the Cohen’s *d* coefficient was high, which suggests a strong effect of the presence of meta- and hypolimnetic hypoxia and the presence of both a higher temperature in the epilimnion and meta- and hypolimnetic hypoxia on *Daphnia* distribution.

### Comparison of fish distribution between each experimental treatment and the control

The comparison of the fish distribution separately for each of the sectors revealed no significant differences between the control and experimental treatment in E2 and E4 (Student’s *t*-test, Table II; Fig. 3). In E1, differences were found in the two upper sectors. The distribution in sector 1 was greater in the control, while the distribution in sector 2 was greater in the experimental treatment (Student’s *t*-test, Table II; Fig. 3), suggesting that the fish foraged slightly deeper in the water column at the higher epilimnetic temperature in comparison to the control. In E3, differences were also found in the two upper sectors. The distribution in sector 1 was greater in the experimental treatment and greater in the control in sector 2 (Student’s *t*-test, Table II; Fig. 3), suggesting that the fish selected depths slightly closer to the surface in the presence of both a higher temperature in the epilimnion and meta- and hypolimnetic hypoxia in comparison to the control. In each of the four comparisons where significant differences were found (two in E1 and two in E3), the Cohen’s *d* coefficient was high, which suggests a strong effect of the presence of meta- and hypolimnetic hypoxia and the presence of both a higher temperature in the epilimnion and meta- and hypolimnetic hypoxia on fish distribution.

### Comparison of the predation rate of the fish between each of the experimental treatments and the control as well as between the experimental treatments

**Fig. 4:**
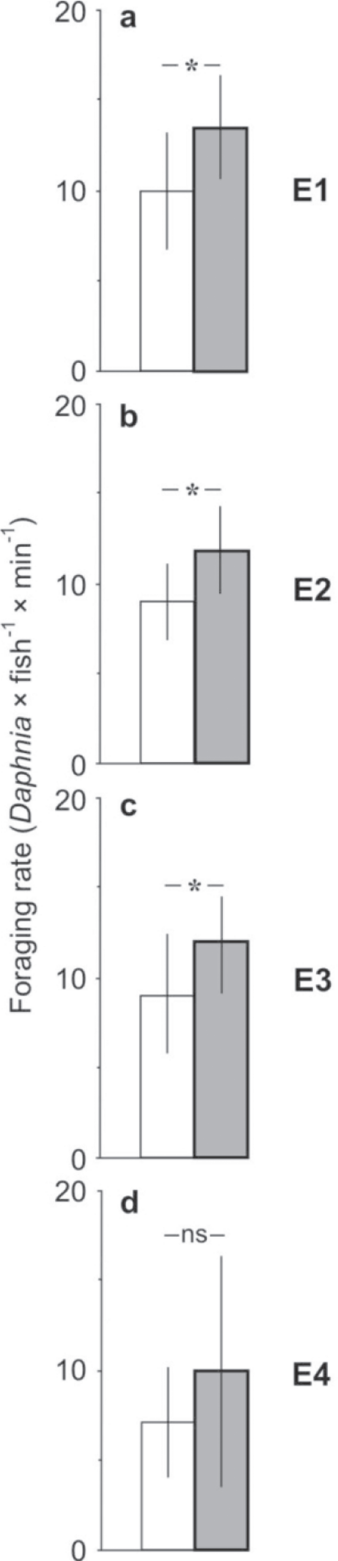
Predation rate of planktivorous fish (mean ± 1 SD) on Daphnia in the control (white bars) and in one of the four experimental treatments (gray bars): (1) elevated temperature in the epilimnion (**a**), (2) the presence of hypoxia in the meta- and hypolimnion (**b**), (3) both higher temperature in the epilimnion and hypoxia in the meta- and hypolimnion (**c**) and (4) Daphnia and fish acclimated to hypoxia before the experiments (**d**).

The predation rate of the fish was greater in the higher temperature in the epilimnion (Student’s *t*-test, Table III; Fig. 4a), in meta- and hypolimnetic hypoxia (Student’s *t*-test, Table III; Fig. 4b) and in both the higher temperature in the epilimnion and meta- and hypolimnetic hypoxia (Student’s *t*-test, Table III; Fig. 4c). The effect of acclimating the fish and *Daphnia* to hypoxia (in E4) was not significant (Student’s t-test, Table III; Fig. 4d).

The predation rate significantly differed in the experimental treatments compared to the control (*F*_3,37_ = 22.78; *P* < 0.001, one-way ANOVA). The only difference in the predation rate among the experimental treatments was observed between E4 and the remaining types of experiments (E1–3; *P* < 0.001, LSD *post-hoc* test).

## DISCUSSION

Our study confirmed the general hypothesis that an increased temperature in the epilimnion and the presence of hypoxia in the meta- and hypolimnion (which is often accompanied by an elevated epilimnetic te mperature) results in an increased predation rate of fish. The results suggest that the effect seems to be due to the greater foraging rate and the increased mean depth selected by the fish rather than by the *Daphnia* at the elevated epilimnetic temperature and due to the decreased mean depth selected by *Daphnia* rather than by the fish in the presence of meta- and hypolimnetic hypoxia. Therefore, the results suggest that both alterations could be responsible for decreasing densities and mean body size in zooplankton populations and communities at elevated ambient temperatures, either spatially (with increasing temperature along altitude and latitude) or temporally (during summer and with increasing temperature due to ongoing climate change).

**Table II:** *Results of the Student’s* t*-test for independent samples comparing fish distribution in each of the two upper sectors (0–50 and 50–110 cm) in the water column between the experimental treatment and control in each of the four types of experiments: (E1) control and treatment with higher temperature in the epilimnion, (E2) control and treatment with a low oxygen concentration (hypoxia) in the meta- and hypolimnion, (E3) control and treatment with both higher temperature in the epilimnion and hypoxia and (E4) control with* Daphnia *and fish acclimated to normoxia and treatment with* Daphnia *and fish acclimated to hypoxia*

Exp. type	Sector # (depth range in centimeters)	Exp. treatment (*n* = 10)		Control (*n* = 10)				95% CI		
		M	SD	M	SD	*t*	*P*	LL	UL	Cohen’s *d*
E1	1 (0–50)	76.60	10.12	72.05	7.57	1.14	0.270	−3.85	12.95	0.51
	2 (50–90)	23.40	10.12	27.95	7.57	1.14	0.270	−3.85	12.95	0.51
E2	1 (0–50)	67.30	14.46	81.37	6.57	−2.80	**0.012**	−24.62	−3.51	1.25
	2 (50–90)	33.70	14.90	17.63	5.29	3.21	**0.005**	5.56	26.57	1.44
E3	1 (0–50)	82.79	5.97	71.33	9.50	3.23	**0.005**	4.00	18.91	1.44
	2 (50–90)	17.21	5.97	28.67	9.50	−3.23	**0.005**	−18.91	−4.00	1.44
E4	1 (0–50)	79.00	12.87	78.50	10.01	0.10	0.924	−10.33	11.33	0.04
	2 (50–90)	21.00	12.87	21.50	10.01	−0.10	0.924	−11.33	10.33	0.04

Significant differences are marked in bold. M – mean; SD – standard deviation; *t* – Student’s *t*-test result; *P* – significance; CI *–* confidence interval; LL – lower limit; UL – upper limit.

**Table III:** *Results of the Student’s* t*-test for independent samples comparing the predation rate of planktivorous fish on* Daphnia *between the experimental treatment and control in each of the four types of experiments: (E1) control and treatment with higher temperature in the epilimnion, (E2) control and the treatment with a low oxygen concentration (hypoxia) in the meta- and hypolimnion, (E3) control and treatment with both higher temperature in the epilimnion and hypoxi, and (E4) control with* Daphnia *and fish acclimated to normoxia and treatment with* Daphnia *and fish acclimated to hypoxia*

Exp. type	Exp. treatment (*n* = 10)		Control (*n* = 10)				95% CI		
	M	SD	M	SD	*t*	*P*	LL	UL	Cohen’s *d*
E1	13.42	2.59	9.30	2.61	3.54	0.002	1.68	6.56	1.59
E2^a^	13.44	2.43	10.23	2.39	3.12	0.005	1.07	5.36	1.33
E3	11.86	2.09	8.94	2.07	3.14	0.006	0.97	4.88	1.41
E4	5.26	3.17	3.98	1.65	1.13	0.273	−1.10	3.66	0.51

Significant differences are marked in bold. M – mean; SD – standard deviation; *t* – the result of the Student’s *t*-test; *P* – significance; CI – confidence interval; LL – lower limit; UL – upper limit. ^a^*n* = 11/11.

The results confirmed our first specific hypothesis, since the predation rate of the planktivorous fish was greater at the elevated temperature in the epilimnion than in the control. Although the literature provides numerous experimental evidence that the foraging rate of planktivorous fish increases at elevated temperature (Persson, 1986; Bergman, 1987; Linløkken *et al*., 2010; Gliwicz and Maszczyk, 2016), our study seems to be the first providing direct evidence that this is also the case when both *Daphnia* and fish distribute freely in a gradient of temperature with a warmer epilimnion. The effect seems to be due to both the greater foraging rate at the elevated epilimnetic temperature and the increased mean depth selected by the fish, rather than by the *Daphnia* (i.e. decreased thickness of the metalimnetic refuge), at the elevated epilimnetic temperature, which in turn increased the time spent in the metalimnion, where the density of zooplankton was the highest. Moreover, the results suggest that the impact of increased predation at an elevated temperature in the epilimnion on mortality risk and, in turn, on the population growth rate of the zooplankton, would be greater than the potential effect of temperature on the birth rate (by increasing the metabolic rate), since *Daphnia* resided at a similar mean depth, and thus at a similar temperature in the experimental treatment and in the control.

The results also confirmed the second specific hypothesis, because the predation rate of the fish was lower in the control treatment than under meta- and hypolimnetic hypoxia. The effect seems to be the result of the fact that the presence of meta- and hypolimnetic hypoxia decreased the mean depth selected by *Daphnia* but not by the fish. The greater predation rate of the fish in hypolimnetic hypoxia confirmed the results of outdoor enclosure experiments (Wright and Shapiro, 1990; Field and Prepas, 1997; Chang *et al*., 2013) but contradicts the results from the single indoor experiment (Larsson and Lampert, 2011). Two possible explanations for the opposing results are most likely. The first explanation concerns the differences in the food gradient created in the experiments, where the maximum concentration was in the subsurface layers in our study, while the study of Larsson and Lampert (2011) had a deep chlorophyll maximum. Favorable food conditions for *Daphnia* in the metalimnion would result in increasing the mean depth selected by *Daphnia* and decreasing its mortality risk to fish predation. The second explanation concerns the differences in the *Daphnia* and fish species used in the studies, with medium-bodied *D. longispina* and rudd in our study, and the larger-bodied *Daphnia pulicaria* and ide (*Leuciscus idus)* in the study of Larsson and Lampert (2011). The latter explanation seems likely in the light of observations from numerous studies indicating a great variability in the vulnerability to hypoxia of different *Daphnia* (Heisey and Porter, 1977; Sell, 1998; Wilczyński *et al*., 2019) and fish (Ekau *et al*., 2010) species, with *D. pulicaria* as particularly resistant (Larsson and Lampert, 2012) and ide as a particularly vulnerable fish species to oxygen deficiencies (Mann, 1996).

Although the combined effect of increased temperature in the epilimnion and the presence of hypoxia in the metalimnion on the predation rate was significant, it was not greater than the effect of each of the factors acting separately. Therefore, the results did not confirm our third specific hypothesis. It is worth noting that in the presence of both alterations, the fish migrated upwards, and in the presence of elevated temperature in the epilimnion alone, they migrated downwards compared to the control. This suggests that in our experiments, the presence of metalimnetic hypoxia had a greater impact on the distribution of fish than the presence of elevated epilimnetic temperature.

Also, the results did not confirm our fourth specific hypothesis, since we did not detect an effect of the acclimation of fish and *Daphnia* to hypoxia on the predation rate of fish in the gradient of oxygen concentration. It should be pointed out that fish and *Daphnia* were acclimated to hypoxia only for a single period (2 days) before the experiments and were also kept in normoxia for a single period (1 hour) just before the experiments to neutralize the potential short-lasting negative effects of oxygen deficiency. It cannot be excluded that extending each of the two maintenance periods would have allowed us to obtain a positive or negative effect of acclimation on the predation rates.

Although the results of our study clearly revealed that both alterations (increased temperature in the epilimnion and the presence of hypoxia in the metalimnion) result in increasing the predation rate of planktivorous fish, the interpretation of the results should be treated with caution for at least two general reasons. The first reason is that we only used a single fish species and a single *Daphnia* clone in the experiments; therefore, the experiment ignores the potential importance of inter- and intraspecific genetic variation. It is very likely that using a different combination of species and clones could give a different result, since it is well known that different *Daphnia* species (Stich and Lampert, 1981; Sakwinska and Dawidowicz, 2005) and clones (De Meester, 1993; Michels and De Meester, 2004) and different species of planktivorous fish (Persson, 1986; Bergman, 1987) prefer different combinations of environmental conditions (i.e. light intensity, temperature and oxygen concentration), and in turn different depths in the water column. The second reason is that due to the short term of the experiment, we only assessed the direct (alterations of *Daphnia’s* vertical distribution and therefore, encounter probability with fish), but not the long-lasting (i.e. microevolutionary and evolutionary dynamics, metabolic costs, recruitment of warm-water juvenile fish, bottom-up effects) population effects of increased temperature in the epilimnion and the presence of hypoxia in the metalimnion. Since it has been revealed that local adaptations of *Daphnia* populations to low oxygen (Weider and Lampert, 1985; Nebeker *et al*., 1992; Seidl, Paul and Pirow, 2005) and high temperatures (Scheiner and Yampolsky, 1998; Van Doorslaer *et al*., 2009; Van Doorslaer *et al*., 2010; Geerts *et al*., 2015) are rapid, microevolutionary and evolutionary responses, this may potentially alter the responses of *Daphnia* populations in their vertical distribution to fish predation when confronted with increased temperature and/or decreased oxygen availability. Moreover, an elevated temperature in the epilimnion and the presence of hypoxia in the metalimnion may also determine population growth through other mechanisms over longer time scales, including metabolic costs (Somero 2002; Wiklund, Sundelin 2004; Ekau *et al*., 2010), recruitment of warm-water juvenile fish (Gauthier *et al*., 2014) and several bottom-up effects (Sastri *et al*., 2014). Due to the aforementioned limitations, the short-term responses observed in our study will not necessarily represent the entire dynamics of actual field populations.

## CONCLUSION

To conclude, several mechanisms responsible for the increased effect of fish predation have been proposed to explain the decreasing density and mean body size of zooplankton populations and communities. For instance, there is general agreement that seasonal variation in body size of the communities is due to the appearance of high densities of juvenile fish in early summer (Sommer *et al*., 1986). A similar explanation has been proposed for the latitudinal changes in the size structure of zooplankton communities, that is, the pattern is due to greater densities of small omnivorous fish (with a short lifespan, early maturation, high reproductive frequencies and a prolonged spawning season) in subtropical and tropical latitudes, which efficiently forage on zooplankton (Jeppesen *et al*., 2010; Iglesias *et al*., 2011). Although the results we obtained should be treated with caution, they suggest that changes in the gradients of the physical parameters in the water at an elevated temperature would be also responsible for the increased effect of fish predation on zooplankton populations and communities in warm lakes.

## Supplementary Material

Appendix_1_fbz048
