# Supplementary material for: Combined effects of elevated epilimnetic temperature and metalimnetic hypoxia on the predation rate of planktivorous fish
Source: J Plankton Res. 2019 Oct 9;41(5):709–22. doi: 10.1093/plankt/fbz048 (PMC6862932; doi:10.1093/plankt/fbz048)
Supplement: Appendix_1_fbz048 [file appendix_1_fbz048.doc]

**Appendix 1.**

**Table I**. Basic descriptive statistics for *Daphnia* distribution in each of 8 sectors of the water column in the experimental treatment (E) and in the control (Cont.) for the data obtained in the first type of the experiments (E1), in which the experimental treatment differed from the control by a higher temperature in the epilimnion.

| Sector #  (depth range in cm) | Treatment | *M* | *Me* | *SD* | *Sk.* | *Kurt.* | *Min* | *Max* | *S-W* | *p* |
| --- | --- | --- | --- | --- | --- | --- | --- | --- | --- | --- |
| 1 (0-20) | E | 11.79 | 8.73 | 7.70 | 1.44 | 1.85 | 3.65 | 29.00 | 0.85 | 0.064 |
|  | Cont. | 12.76 | 8.13 | 10.61 | 1.19 | 0.06 | 4.11 | 32.11 | 0.80 | 0.013 |
| 2 (20-40) | E | 12.45 | 11.86 | 7.10 | 0.84 | 0.73 | 2.85 | 27.12 | 0.94 | 0.600 |
|  | Cont. | 10.23 | 8.99 | 6.75 | 1.43 | 2.24 | 4.18 | 25.69 | 0.86 | 0.068 |
| 3 (40-60) | E | 18.24 | 18.73 | 8.05 | -0.12 | -1.67 | 7.46 | 28.79 | 0.91 | 0.259 |
|  | Cont. | 15.04 | 13.86 | 10.96 | 0.92 | 0.27 | 4.18 | 37.00 | 0.90 | 0.212 |
| 4 (60-80) | E | 24.76 | 25.31 | 10.77 | -0.17 | -1.61 | 10.00 | 36.97 | 0.89 | 0.164 |
|  | Cont. | 25.61 | 25.04 | 13.73 | -0.03 | -1.68 | 7.00 | 41.70 | 0.89 | 0.186 |
| 5 (80-100) | E | 23.07 | 22.92 | 10.45 | -0.13 | -1.02 | 6.78 | 36.97 | 0.96 | 0.730 |
|  | Cont. | 26.07 | 25.04 | 13.19 | 0.01 | -1.67 | 9.09 | 41.70 | 0.89 | 0.155 |
| 6 (100-120) | E | 5.69 | 6.16 | 3.41 | 0.25 | -1.22 | 1.85 | 11.17 | 0.90 | 0.240 |
|  | Cont. | 6.37 | 5.75 | 5.43 | 1.08 | 1.31 | 0.00 | 18.18 | 0.91 | 0.266 |
| 7 (120-140) | E | 2.39 | 1.85 | 2.13 | 1.16 | 1.04 | 0.00 | 6.78 | 0.84 | 0.045 |
|  | Cont. | 1.60 | 2.03 | 1.07 | -0.48 | -0.99 | 0.00 | 3.03 | 0.90 | 0.196 |
| 8 (140-160) | E | 0.00 | 0.00 | 0.00 | - | - | 0.00 | 0.00 | - | - |
|  | Cont. | 0.00 | 0.00 | 0.00 | - | - | 0.00 | 0.00 | - | - |

*M* – mean; *Me* – median; *SD* – standard deviation; *Sk.* – skewness*; Kurt. – ­*kurtosis; *Min* and *Max*–lowest and highest value of the distribution; *S-W* – Shapiro-Wilk test result; *p* – significance

**Table II**. The basic descriptive statistics for *Daphnia* distribution in each of 8 sectors of the water column in the experimental treatment (E) and in the control (Cont.) for the data obtained in the second type of the experiments (E2), in which the experimental treatment differed from the control by a low oxygen concentration (hypoxia) in the meta- and hypolimnion.

| Sector #  (depth range in cm) | Treatment | *M* | *Me* | *SD* | *Sk.* | *Kurt.* | *Min* | *Max* | *S-W* | *p* |
| --- | --- | --- | --- | --- | --- | --- | --- | --- | --- | --- |
| 1 (0-20) | E | 53.03 | 61.80 | 20.84 | -0.40 | -2.04 | 25.50 | 72.63 | 0.79 | 0.012 |
|  | Cont. | 33.32 | 36.27 | 10.98 | -0.30 | -1.19 | 17.36 | 48.20 | 0.92 | 0.391 |
| 2 (20-40) | E | 23.10 | 18.81 | 8.60 | 0.49 | -1.77 | 13.00 | 35.00 | 0.84 | 0.046 |
|  | Cont. | 23.67 | 19.78 | 10.83 | -0.07 | -0.83 | 4.89 | 39.00 | 0.91 | 0.291 |
| 3 (40-60) | E | 10.88 | 9.94 | 6.66 | 0.15 | -2.19 | 3.96 | 20.00 | 0.82 | 0.026 |
|  | Cont. | 13.92 | 14.25 | 1.83 | 1.00 | 1.52 | 12.00 | 17.93 | 0.87 | 0.087 |
| 4 (60-80) | E | 7.71 | 5.00 | 6.07 | 0.84 | -0.73 | 1.10 | 18.00 | 0.87 | 0.105 |
|  | Cont. | 10.00 | 9.87 | 6.89 | 0.17 | -0.30 | 0.00 | 22.48 | 0.95 | 0.628 |
| 5 (80-100) | E | 2.79 | 1.15 | 4.21 | 1.75 | 1.89 | 0.00 | 12.00 | 0.69 | 0.001 |
|  | Cont. | 9.93 | 7.94 | 8.69 | 1.45 | 1.73 | 1.70 | 29.00 | 0.82 | 0.024 |
| 6 (100-120) | E | 2.02 | 1.15 | 3.02 | 1.88 | 2.70 | 0.00 | 9.00 | 0.69 | 0.001 |
|  | Cont. | 6.06 | 7.25 | 5.38 | 0.59 | -0.23 | 0.00 | 16.29 | 0.90 | 0.199 |
| 7 (120-140) | E | 1.64 | 0.39 | 2.74 | 1.77 | 1.73 | 0.00 | 7.50 | 0.63 | <0.001 |
|  | Cont. | 2.58 | 1.47 | 2.58 | 1.02 | -0.31 | 0.00 | 7.44 | 0.83 | 0.029 |
| 8 (140-160) | E | 0.29 | 0.19 | 0.41 | 1.95 | 4.53 | 0.00 | 1.30 | 0.71 | 0.001 |
|  | Cont. | 0.60 | 0.69 | 0.61 | 0.63 | -0.51 | 0.00 | 1.73 | 0.86 | 0.072 |

*M* – mean; *Me* – median; *SD* – standard deviation; *Sk.* – skewness*; Kurt. – ­*kurtosis; *Min* and *Max*– lowest and highest value of the distribution; *S-W* – Shapiro-Wilk test result; *p* – significance

**Table III**. The basic descriptive statistics for *Daphnia* distribution in each of 8 sectors of the water column in the experimental treatment (E) and in the control (Cont.) for the data obtained in the third type of the experiments (E3), in which the experimental treatment differed from the control by both a higher temperature in the epilimnion and hypoxia in the meta- and hypolimnion.

| Sector #  (depth range in cm) | Treatment | *M* | *Me* | *SD* | *Sk.* | *Kurt.* | *Min* | *Max* | *S-W* | *p* |
| --- | --- | --- | --- | --- | --- | --- | --- | --- | --- | --- |
| 1 (0-20) | E | 16.91 | 15.55 | 8.50 | 0.50 | -0.98 | 6.23 | 31.43 | 0.94 | 0.592 |
|  | Cont. | 16.46 | 17.72 | 6.88 | -0.46 | -0.79 | 3.96 | 25.00 | 0.94 | 0.519 |
| 2 (20-40) | E | 27.00 | 24.87 | 8.75 | 0.48 | -1.42 | 16.39 | 39.79 | 0.89 | 0.152 |
|  | Cont. | 16.35 | 14.70 | 8.05 | 1.44 | 1.08 | 9.17 | 32.88 | 0.79 | 0.011 |
| 3 (40-60) | E | 17.39 | 16.86 | 5.59 | 0.55 | -0.03 | 9.49 | 27.49 | 0.95 | 0.611 |
|  | Cont. | 14.61 | 13.87 | 7.96 | 0.63 | 0.16 | 2.38 | 27.99 | 0.91 | 0.255 |
| 4 (60-80) | E | 15.81 | 17.36 | 6.66 | -0.31 | -1.07 | 4.74 | 25.00 | 0.96 | 0.739 |
|  | Cont. | 14.68 | 12.95 | 6.90 | 1.61 | 2.52 | 8.22 | 30.65 | 0.83 | 0.035 |
| 5 (80-100) | E | 12.37 | 12.95 | 4.55 | -0.21 | -0.91 | 4.74 | 18.96 | 0.97 | 0.912 |
|  | Cont. | 16.04 | 14.35 | 7.48 | 0.79 | -0.08 | 7.50 | 30.65 | 0.93 | 0.492 |
| 6 (100-120) | E | 5.46 | 5.72 | 3.71 | 0.25 | -0.20 | 0.00 | 12.19 | 0.98 | 0.960 |
|  | Cont. | 11.02 | 11.52 | 6.65 | -0.21 | -1.07 | 0.91 | 20.00 | 0.95 | 0.715 |
| 7 (120-140) | E | 4.70 | 5.38 | 4.31 | 0.30 | -1.02 | 0.00 | 12.19 | 0.90 | 0.236 |
|  | Cont. | 7.18 | 8.08 | 4.11 | -1.12 | 0.28 | 0.00 | 11.97 | 0.84 | 0.049 |
| 8 (140-160) | E | 2.80 | 1.97 | 3.29 | 1.38 | 1.15 | 0.00 | 9.76 | 0.82 | 0.028 |
|  | Cont. | 3.04 | 2.45 | 2.74 | 0.35 | -1.38 | 0.00 | 7.14 | 0.89 | 0.161 |

*M* – mean; *Me* – median; *SD* – standard deviation; *Sk.* – skewness*; Kurt. – ­*kurtosis; *Min* and *Max*–lowest and highest value of the distribution; *S-W* – Shapiro-Wilk test result; *p* – significance

**Table IV**. The basic descriptive statistics for *Daphnia* distribution in each of 8 sectors of the water column in the experimental treatment (E) and in the control (Cont.) for the data obtained in the fourth type of the experiments (E4), in which the gradients of temperature and oxygen concentration (with meta- and hypolimnetic hypoxia) were the same in both the control and the experimental treatment, but *Daphnia* and fish were acclimated to normoxia in the control and to hypoxia in the experimental treatment two days before the experiments.

| Sector #  (depth range in cm) | Treatment | *M* | *Me* | *SD* | *Sk.* | *Kurt.* | *Min* | *Max* | *S-W* | *p* |
| --- | --- | --- | --- | --- | --- | --- | --- | --- | --- | --- |
| 1 (0-20) | E | 22.18 | 22.06 | 6.32 | -0.89 | 0.52 | 9.09 | 28.13 | 0.87 | 0.088 |
|  | Cont. | 24.60 | 25.93 | 8.86 | -0.83 | 0.68 | 7.32 | 35.71 | 0.86 | 0.070 |
| 2 (20-40) | E | 16.89 | 17.60 | 5.44 | -0.89 | 0.09 | 6.06 | 21.88 | 0.87 | 0.098 |
|  | Cont. | 16.65 | 18.52 | 4.32 | -0.29 | 0.01 | 10.00 | 24.00 | 0.88 | 0.146 |
| 3 (40-60) | E | 12.15 | 9.83 | 5.58 | 2.67 | 7.48 | 9.09 | 27.27 | 0.60 | <0.001 |
|  | Cont. | 12.04 | 9.88 | 5.81 | 0.18 | -1.92 | 4.00 | 18.52 | 0.82 | 0.025 |
| 4 (60-80) | E | 13.25 | 13.69 | 3.29 | -0.11 | -1.41 | 8.51 | 18.18 | 0.90 | 0.233 |
|  | Cont. | 15.90 | 11.81 | 7.66 | 1.32 | 1.21 | 8.00 | 32.50 | 0.84 | 0.039 |
| 5 (80-100) | E | 13.59 | 12.50 | 3.25 | -0.27 | 1.10 | 7.14 | 19.15 | 0.92 | 0.321 |
|  | Cont. | 7.81 | 10.00 | 7.00 | -0.22 | -2.09 | 0.00 | 16.00 | 0.89 | 0.015 |
| 6 (100-120) | E | 10.04 | 9.38 | 3.18 | 0.30 | 0.99 | 4.26 | 15.15 | 0.84 | 0.044 |
|  | Cont. | 12.19 | 11.11 | 3.45 | 1.86 | 2.40 | 10.00 | 20.00 | 0.66 | <0.001 |
| 7 (120-140) | E | 6.95 | 5.88 | 4.18 | 0.73 | -0.82 | 3.13 | 14.28 | 0.85 | 0.061 |
|  | Cont. | 7.24 | 6.67 | 3.66 | 0.02 | -2.15 | 2.86 | 11.11 | 0.81 | 0.018 |
| 8 (140-160) | E | 4.36 | 3.03 | 4.87 | 0.93 | 0.18 | 0.00 | 14.28 | 0.85 | 0.061 |
|  | Cont. | 2.99 | 3.28 | 2.09 | 0.42 | 1.46 | 0.00 | 7.32 | 0.88 | 0.137 |

*M* – mean; *Me* – median; *SD* – standard deviation; *Sk.* – skewness*; Kurt. – ­*kurtosis; *Min* and *Max*–the lowest and highest value of the distribution; *S-W* – Shapiro-Wilk test result; *p* – significance

**Table V**. The basic descriptive statistics for fish distribution in the two upper sectors of the water column (0-50 and 50-110 cm) in the experimental treatment (E) and in the control (Cont.) for the data obtained in each of the four types of experiments (E1-E4).

| Exp. type | Sector #  (depth range in cm) | Treatment | *M* | *Me* | *SD* | *Sk.* | *Kurt.* | *Min* | *Max* | *S-W* | *p* |
| --- | --- | --- | --- | --- | --- | --- | --- | --- | --- | --- | --- |
| E1 | 1 (0-50) | E | 76.60 | 77.50 | 10.12 | -0.30 | -0.98 | 60.00 | 91.00 | 0.96 | 0.746 |
|  |  | Cont. | 72.05 | 70.50 | 7.57 | 0.20 | -1.85 | 62.50 | 83.00 | 0.89 | 0.164 |
|  | 2 (50-90) | E | 23.40 | 22.50 | 10.12 | 0.30 | -0.98 | 9.00 | 40.00 | 0.96 | 0.746 |
|  |  | Cont. | 27.95 | 29.50 | 7.57 | -0.20 | -1.85 | 17.00 | 37.50 | 0.89 | 0.164 |
| E2 | 1 (0-50) | E | 67.30 | 70.00 | 14.46 | -1.97 | 5.84 | 30.00 | 84.00 | 0.75 | 0.004 |
|  |  | Cont. | 81.37 | 80.00 | 6.57 | 0.04 | -0.15 | 70.00 | 91.00 | 0.95 | 0.614 |
|  | 2 (50-90) | E | 33.70 | 30.00 | 14.90 | 1.70 | 4.00 | 16.00 | 70.00 | 0.83 | 0.035 |
|  |  | Cont. | 17.63 | 20.00 | 5.29 | -0.75 | -0.30 | 9.00 | 25.00 | 0.88 | 0.126 |
| E3 | 1 (0-50) | E | 82.79 | 84.00 | 5.97 | -0.28 | -1.55 | 74.00 | 90.00 | 0.91 | 0.298 |
|  |  | Cont. | 71.33 | 70.14 | 9.50 | -0.07 | -1.48 | 59.00 | 85.00 | 0.90 | 0.216 |
|  | 2 (50-90) | E | 17.21 | 16.00 | 5.97 | 0.28 | -1.55 | 10.00 | 26.00 | 0.91 | 0.298 |
|  |  | Cont. | 28.67 | 29.86 | 9.50 | 0.07 | -1.48 | 15.00 | 41.00 | 0.90 | 0.216 |
| E4 | 1 (0-50) | E | 79.00 | 70.00 | 12.87 | 1.01 | -0.81 | 70.00 | 100.00 | 0.71 | 0.001 |
|  |  | Cont. | 78.50 | 77.50 | 10.01 | 1.24 | 1.14 | 70.00 | 100.00 | 0.83 | 0.033 |
|  | 2 (50-90) | E | 21.00 | 30.00 | 12.87 | -1.01 | -0.81 | 0.00 | 30.00 | 0.71 | 0.001 |
|  |  | Cont. | 21.50 | 22.50 | 10.01 | -1.24 | 1.14 | 0.00 | 30.00 | 0.83 | 0.033 |

*M* – mean; *Me* – median; *SD* – standard deviation; *Sk.* – skewness*; Kurt. – ­*kurtosis; *Min* and *Max* – lowest and highest value of the distribution; *S-W* – Shapiro-Wilk test result; *p* – significance

**Table VI.** The basic descriptive statistics for the predation rate in the experimental treatment (E) and in the control (Cont.) for the data obtained in each of the four types of experiments (E1-E4).

| Exp. type | Treatment | *M* | *Me* | *SD* | *Sk.* | *Kurt.* | *Min* | *Max* | *S-W* | *p* |
| --- | --- | --- | --- | --- | --- | --- | --- | --- | --- | --- |
| E1 | E | 13.42 | 14.35 | 2.59 | -0.83 | -0.59 | 9.15 | 16.10 | 0.87 | 0.090 |
|  | Cont. | 9.30 | 9.15 | 2.61 | -0.07 | -0.42 | 4.93 | 13.10 | 0.96 | 0.806 |
| E2 | E | 13.44 | 14.70 | 2.43 | -0.93 | -0.62 | 9.07 | 16.10 | 0.86 | 0.052 |
|  | Cont. | 10.23 | 11.20 | 2.39 | -0.23 | -0.77 | 6.75 | 14.20 | 0.91 | 0.215 |
| E3 | E | 11.86 | 11.50 | 2.09 | 0.90 | 0.85 | 9.07 | 16.20 | 0.94 | 0.591 |
|  | Cont. | 8.94 | 9.50 | 2.07 | -0.49 | -1.48 | 5.71 | 11.20 | 0.89 | 0.153 |
| E4 | E | 5.26 | 5.32 | 3.17 | 0.70 | 0.88 | 1.25 | 11.85 | 0.92 | 0.396 |
|  | Cont. | 3.98 | 3.71 | 1.65 | 0.77 | 0.04 | 1.96 | 7.05 | 0.92 | 0.353 |

*M* – mean; *Me* – median; *SD* – standard deviation; *Sk.* – skewness*; Kurt. – ­*kurtosis; *Min* and *Max*–lowest and highest value of the distribution; *S-W* – Shapiro-Wilk test result; *p* – significance
